# Uterine torsion as an elusive obstetrical emergency in pregnancy: is there an association between gravid uterus torsion and Ehlers–Danlos syndrome?: a case report

**DOI:** 10.1186/s13256-022-03409-4

**Published:** 2022-05-17

**Authors:** Seyedeh Noushin Ghalandarpoor-Attar, Seyedeh Mojgan Ghalandarpoor-Attar

**Affiliations:** 1 Obstetrics and Gynecology Department, Baqiyatallah Hospital, School of Medicine, Baqyiatallah University of Medical Sciences, Tehran, Iran; 2 Obstetrics and Gynecology Department, Baharloo Hospital, Tehran University of Medical Sciences, Tehran, Iran

**Keywords:** Uterine torsion, Pregnancy, Placenta abruption, Connective tissue disorder, Ehlers–Danlos syndrome

## Abstract

**Background:**

Timely diagnosis of uterine torsion can lead to acceptable maternal and fetal outcomes. This article presents the case of a 42-year-old pregnant woman, diagnosed with a rare 270° uterine torsion, in whom proper management led to good maternal outcomes but, unfortunately, severe prematurity and metabolic acidosis led to neonatal death. Moreover, the mother was clinically suspected for Ehlers–Danlos syndrome.

**Case presentation:**

In December 2020, a 42-year-old pregnant Iranian woman, Gravid 3 para2 live2, at 30 weeks of gestation presented to the obstetric emergency department of Vali-Asr Hospital (Birjand, Iran) suffering from acute severe generalized abdominal pain, nausea, vomiting, and dizziness while she was hemodynamically unstable. After resuscitation, owing to persistent fetal bradycardia on fetal heart rate monitoring, she underwent an emergency cesarean section. Infra-umbilical midline skin incision was made, and when the abdominal cavity was opened, owing to abnormal appearance of the uterus, we further investigated the abdominopelvic cavity. Surprisingly, the uterus was dextrorotated by 270°. After uterine detorsion through a Kerr incision, a nonvigorous male baby was born with severe metabolic acidosis that led to his death soon after birth. Interestingly, we could find no predisposing factors such as pelvic abnormalities during surgery. Nevertheless, as her postoperative detailed physical examination revealed skin hyperextensibility, joint laxity, pelvic organ prolapse, and trivial exophthalmos, connective tissue disorders, mainly Ehlers–Danlos syndrome, were suspected. Unfortunately, for significant financial, cultural, and religious reasons, the patient refused to undergo further investigations. Additionally, despite severe congested uterus and subsequent uterine atony, timely diagnosis and anatomical correction of the gravid uterus before uterine incision prevented iatrogenic complications. The mother was discharged 2 days later without any postpartum complications.

**Conclusion:**

Although uterine torsion is an extremely rare condition during pregnancy, based on severe associated maternal and perinatal complications, it is important to take this diagnosis into consideration as an differential diagnosis. Moreover, connective tissue disorders seem to be a potential risk factor for uterine torsion, although further studies on this subject are required.

## Background

Uterine torsion is defined as rotation of the uterus around its long axis greater than 45°, and it mainly occurs during pregnancy [1]. Rotation degree varies widely, from 45° to 180°, and dextrorotation is much more frequent [2–5]. Surprisingly, most obstetricians and gynecologists may encounter this obstetrical emergency only once in their lives [6]. On the other hand, although uterine torsion complications in pregnancy depend mainly on degree of rotation, gestational age, and time elapsed to diagnosis [7–9], in rotations exceeding 180°, maternal and fetal mortality rates have been reported up to 36% and 71%, respectively [7]. Owing to nonspecific and vague symptoms that can mimic other obstetrical complications, mainly placental abruption, its diagnosis is elusive and is almost always postponed to the operating room [7]. Delayed diagnosis can also result in serious iatrogenic injuries to the uterus and/or adjacent organs, especially the urinary system. Hence, strong clinical suspicion, especially when the patient has an underlying risk factor, can reduce both maternal and fetal mortality and morbidity [10–15]. Various conditions have been mentioned as predisposing factors, but to the best of our knowledge, there is no report on uterine torsion being associated with Ehlers–Danlos syndrome (EDS) as a leading cause. In this current paper, we present a case of uterine torsion in a clinically suspected pregnant woman with EDS at 30 weeks of gestation.

## Case presentation

A 42-year-old pregnant Iranian woman, Gravid 3 para2 live2, at 30 weeks of gestation was referred to the obstetric emergency department of Vali-Asr Hospital (Birjand, Iran) by the emergency service in December 2020. She was suffering from acute-onset severe abdominal pain followed by nausea, vomiting, hypotension, and dizziness. She had two healthy children, her previous pregnancies were uneventful, and she declared no postpartum complications during her previous gestations. She had no history of cigarette smoking or alcohol consumption, and her marriage was consanguineous. Moreover, she was living in a deprived remote village around Birjand City (Southern Khorasan Province, Iran) with very low socioeconomic status.

Upon her arrival, her body mass index, blood pressure, pulse rate, respiratory rate, oral temperature, and peripheral oxygen saturation in room air were 22.6 kg/m^2^, 70 mmHg, 130 beats per minute, 14 breaths per minute, 36.9 °C, and 98% ,respectively. Her skin and mucosal membranes were completely pale, and urinary output was less than 50 cc per hour. She was lethargic, but neurological examination did not reveal any focal neurological deficit and corneal reflex was symmetrically present in both eyes. Obstetrical examination revealed increased fundal height in excess of a term uterus and also uterine hypertonicity. No evidence of ruptured membranes or external vaginal bleeding was evident. Although on digital vaginal examination the cervix was too high posteriorly positioned to be touched by digits, it seemed unripe and closed. Moreover, continuous fetal heart rate monitoring revealed fetal bradycardia.

As the patient was hemodynamically unstable, resuscitation was initiated with a bolus of 2 L Ringer’s lactate solution immediately and blood tests were sent. Two units of packed cells were cross-matched. After stabilization, owing to persistent fetal bradycardia, as low as 80 beats per minute, she was emergently transferred to the operating room for cesarean section delivery by impression of massive occult placental abruption. Under general anesthesia, the abdominal cavity was accessed through a low midline incision. We encountered with lateral wall of the uterus and left broad ligament. As the uterus was levorotated approximately 90°, a severely congested dark-purple uterus with many dilated tortuous vessels on its lower segment was seen and no uterovesical peritoneal reflex could be identified. Subsequently manual exploration revealed an additional 180° uterine dextrotorsion. Hence, abdominal wall skin incision was expanded to upper umbilical level and the uterus was detorted. Subsequently, a male baby weighing 1150 g was born through a Kerr uterine incision. Unfortunately, the 1-minute Apgar score was 0 and resuscitation was unsuccessful. Umbilical cord blood gas analysis showed severe metabolic acidosis (pH 6.95) and base excess of 16 mmol/L.

It is worth noting that, after detorsion, maternal central and peripheral circulation began to improve rapidly and myometrial layer color changed to near normal, but when the uterine wound was closed, the uterus became atonic. Uterotonic agents (0.2 mg of intramuscular methylergonovine plus 30 international units of intravenous oxytocin)were administered, and owing to moderate anemia (hemoglobin of 9.8 g/dL) detected in her initial laboratory tests, 2 units of packed cells were transfused. Platelet count, coagulation study (prothrombin time, partial prothrombin time, and fibrinogen level), and liver and kidney function tests were all unremarkable. Fortunately, despite anemia, all other laboratory tests, including serum electrolytes, serum aminotransferase, serum creatinine, and coagulation study, were within normal limits post-operation period, and her hemoglobin level reached 10 g/dL.

Interestingly, we could not find any pelvic abnormalities such as congenital anomalies, uterine fibroids, pelvic or adnexal masses, adhesion bands, fetal malpresentation, or any report of vigorous fetal movements prior to symptom onset. However, parietal peritoneum and broad ligaments seemed too loose. In the postoperative period, physical examination and detailed history were taken. No additional significant data were found out except for moderate low back pain; magnetic resonance imaging had revealed lumbar disc herniation 2 years ago. Moreover, her height and body mass index were 1.7 m and 22.6 kg/m^2^, respectively; she showed increased skin elasticity, joint hypermobility in upper extremities, and trivial exophthalmos. On vaginal examination based on pelvic organ prolapse quantification (POP-Q) classification, third-grade anterior and second-grade posterior compartment prolapse were evident; vaginal wall had increased elasticity, and no episiotomy scar or perineal lacerations were observed. In fact, no episiotomy had been made in either vaginal birth, and her previous newborns weighed nearly 3 kg. She received low-molecular-weight heparin 40 mg subcutaneously daily after 24 hours postoperation. On the basis of these systemic manifestations, connective tissue disorders, mainly Ehlers–Danlos, were highly suspected. However, as she was illiterate and living in a remote deprived village, she refused any further investigation, and on the second day postpartum, she was discharged from the hospital on her own request without any complication and was recommended to continue thromboprophylaxis for an additional 7 days. Subsequently, skin sutures were removed on the 12th postoperative day, and she did not come back thereafter. Unfortunately, we were not even able to contact her via phone to have an interview-based follow-up visit. It should be emphasized that, despite refusing to have her pictures taken and undergo further assessments, she gave us informed consent to publish this information.

## Discussion

Here we presented a rare case of 270° gravid uterine torsion manifesting with hemodynamic instability and a clinical picture mimicking extensive occult placental abruption but without evidence of obvious coagulopathy. The patient had systemic clinical features of connective tissue disorders, mainly Ehlers–Danlos syndrome; however, definite diagnosis of Ehlers–Danlos could not be made as the patient refused genetic testing. In summary, the degree of uterine torsion, the shock status despite lack of external or internal bleeding, and accompanying clinical findings in favor of Ehlers–Danlos were all interesting points to be considered.

In fact, uterine torsion is an extremely rare event, especially in nonpregnant women, and often occurs in uncomplicated pregnancies, most commonly in the third trimester. It is associated with serious maternal and perinatal outcomes, and mortality rate reaches up to 12–18% [16–23]. In line with this, in the presented case, uterine torsion occurred in the 30th week of gestation and led to neonatal death. The precise etiology is not well known, but presence of uterine myomas, congenital anomalies, pelvic or adnexal masses or adhesions, abnormal fetal presentation, vigorous fetal movements, and abdominal trauma and high-ordered parities account for 70–84% of uterine torsions [24–27]. As mentioned before, relevant systemic signs and symptoms indicate that connective tissue disorders, most likely Ehlers–Danlos syndrome (EDS), are the only potential leading cause in our patient. There are 13 subtypes of EDS according to the International EDS Consortium, and the very rare vascular subtype predisposes pregnant women to more significant and life-threatening complications such as arterial dissection and/or rupture and uterine rupture [28], whereas pregnant women with hypermobile or classic subtypes of EDS are more susceptible to obstetrical complications such as pelvic organ prolapse, postpartum incontinence, abnormal wound healing, and atrophic scar formation [29, 30]. Still, there is no evidence in the literature of EDS association with gravid uterus torsion. However, owing to the patient’s refusal of further investigations and lack of genetic testing, a definite Ehlers–Danlos diagnosis and subtype specification could not be confirmed. This is the main limitation of our report.

Degree of uterine torsion usually varies between 45° and 180°. Owing to the natural right-side deviation of gravid uterus, dextrorotation is more common and rotation greater than 180° is rare [2–5]. In this presented case, the uterus had rotated in excess of 270°. Uterine torsion manifestation can vary significantly. Mild-to-severe abdominal pain accompanied by neurologic or hypovolemic shock is the most common feature, as seen here. Vaginal bleeding, nausea and vomiting, hemodynamic instability, uterine hypertonicity, increased fundal height, decreased fetal movements, fetal distress, intrauterine fetal death, obstructed labor, and dystocia are other possible manifestations [31, 32]. Surprisingly, our patient had fulfilled almost all these symptoms (hemodynamic instability, nausea and vomiting, severe acute abdominal pain, uterine hypertonicity, increased fundal height, and fetal distress). The diagnosis is difficult and almost always occurs during laparotomy [8, 33], as this patient was transferred to operating room with presumed diagnose of severe occult placental abruption. However, imaging modalities such as ultrasound, computed tomography, or magnetic resonance imaging can facilitate diagnosis before surgery [31, 34, 35]. Nevertheless, because uterine torsion presents as an obstetrical emergency, definite diagnosis is almost always delayed until surgery time and there is not enough time to perform any imaging modalities. In line with this, the patient also manifested shock status and massive occult placental abruption was suspected initially. Moreover, she was referred to the emergency department during night, which makes access to imaging facilities even more difficult in some health centers.

Uterine torsion can obliterate ovarian and/or uterine veins. Subsequently, venous congestion and increased placental cotyledon pressure may result in placental abruption. Furthermore, torsion can compress uterine arterial flow and reduce placental perfusion, leading to fetal distress or even intrauterine death [7]. Surgery is considered the main treatment option, while concomitant cesarean delivery is done in near-term or term pregnancies [32]. Maternal prognosis is usually acceptable, but perinatal mortality is still high [36]. If detorsion is impossible or torsion is not diagnosed before uterine wall incision, lower posterior uterine segment incision may damage uterine vasculature and cause severe adverse events [10–15]. Fortunately, in this patient, definite diagnosis was made before uterine incision. Detorsion was followed by a transverse low anterior uterine wall incision. In rare cases, owing to prolonged or irreversible ischemia and subsequent necrosis of the uterus or adjacent pelvic organs, hysterectomy or oophorectomy may be inevitable [37], although it is worth noting that, in a necrotic uterus, detorsion can cause sudden distributive shock and/or serious coagulopathy as a result of reperfusion syndrome or amniotic fluid emboli [1], as Cook *et al*. stated [38]. Interestingly, as hypovolemic shock was due to obstructed uterine venous flow return in this case, by early uterine detorsion before onset of irreversible ischemia, obvious improvement in both peripheral and central circulation occurred. Furthermore, increased fundal height was due to obliterated venous return without any evidence of concealed retroplacental hematoma formation, and absence of clot formation may explain why no coagulopathy was observed. Fortunately, early laparotomy and detorsion of uterus subsequently resulted in full recovery of uterus circulation and hemodynamic parameters.

## Conclusion

Although uterine torsion is an extremely rare condition among pregnant women, because of its severe associated maternal and perinatal complications, it should be suspected in every patient presenting with its associated vague and nonspecific symptoms. In fact, delayed diagnosis even at the time of surgery, can lead to severe additional iatrogenic complications and even mortality. Moreover, as we could not confirm Ehlers–Danlos diagnosis through genetic testing, we highly recommend further investigations on possible association between Ehlers–Danlos syndrome and/or other connective tissue disorders and uterine torsion. We also strongly suggest physicians pay more attention to this potential risk factor when related systemic signs and symptoms are evident in a patient.

## Data Availability

All relevant data are available at Vali-Asr Hospital, South Khorasan Province. Data sharing is not applicable to this article as no datasets were generated or analyzed during the study.
